# Functional Plant Types Drive Plant Interactions in a Mediterranean Mountain Range

**DOI:** 10.3389/fpls.2016.00662

**Published:** 2016-05-23

**Authors:** Petr Macek, Iván Prieto, Jana Macková, Nuria Pistón, Francisco I. Pugnaire

**Affiliations:** ^1^Faculty of Science, University of South BohemiaČeské Budějovice, Czech Republic; ^2^LINCGlobal, Estación Experimental de Zonas Áridas, Consejo Superior de Investigaciones CientíficasAlmería, Spain; ^3^Centre National de la Recherche Scientifique, Centre d’Ecologie Fonctionnelle et Evolutive UMR 5175, Université de Montpellier – Université Paul Valéry – EPHEMontpellier, France; ^4^Institute of Soil Biology, Biology Centre CASČeské Budějovice, Czech Republic; ^5^Universidade Federal do Rio de JaneiroRio de Janeiro, Brazil

**Keywords:** Biomass allocation, competition, facilitation, functional traits, plant interaction balance, phenotypic plasticity, Sierra Nevada Mountains, water availability

## Abstract

Shrubs have positive (facilitation) and negative (competition) effects on understory plants, the net interaction effect being modulated by abiotic conditions. Overall shrubs influence to great extent the structure of plant communities where they have significant presence. Interactions in a plant community are quite diverse but little is known about their variability and effects at community level. Here we checked the effects of co-occurring shrub species from different functional types on a focal understory species, determining mechanisms driving interaction outcome, and tested whether effects measured on the focal species were a proxy for effects measured at the community level. Growth, physiological, and reproductive traits of *Euphorbia nicaeensis*, our focal species, were recorded on individuals growing in association with four dominant shrub species and in adjacent open areas. We also recorded community composition and environmental conditions in each microhabitat. Shrubs provided environmental conditions for plant growth, which contrasted with open areas, including moister soil, greater N content, higher air temperatures, and lower radiation. Shrub-associated individuals showed lower reproductive effort and greater allocation to growth, while most physiological traits remained unaffected. *Euphorbia* individuals were bigger and had more leaf N under N-fixing than under non-fixing species. Soil moisture was also higher under N-fixing shrubs; therefore soil conditions in the understory may counter reduced light conditions. There was a significant effect of species identity and functional types in the outcome of plant interactions with consistent effects at individual and community levels. The contrasting allocation strategies to reproduction and growth in *Euphorbia* plants, either associated or not with shrubs, showed high phenotypic plasticity and evidence its ability to cope with contrasting environmental conditions.

## Introduction

Plant interactions modulate the structure of plant communities and shape species distribution (Callaway, 2007; Butterfield et al., 2010; Cavieres et al., 2014). Some plant species facilitate establishment and growth of other species through amelioration of physical stress (Moro et al., 1997; le Roux and McGeoch, 2010) or resource supply (Pugnaire et al., 1996; Prieto et al., 2012) while competition may counter facilitation effects determining net interaction outcomes (Tielbörger and Kadmon, 2000; Pugnaire and Luque, 2001; Armas et al., 2011). Indeed, while positive interactions (i.e., facilitation) enhance plant growth, reproduction, and survival of understory species, eventually expanding their distribution range (le Roux et al., 2012), negative interactions (i.e., competition, interference) limit growth and fitness of other species and may even completely exclude them from suitable habitats (Choler et al., 2001; le Roux et al., 2013). Overall, there is now ample evidence of plant interactions enhancing coexistence in plant communities at local (Pugnaire et al., 1996; Holzapfel and Mahall, 1999; Choler et al., 2001; Maestre et al., 2003; le Roux and McGeoch, 2010; Schöb et al., 2012; Tirado et al., 2015) and global scales (Callaway et al., 2002; Kikvidze et al., 2005; Butterfield et al., 2013; He et al., 2013; Cavieres et al., 2014). Yet there is less evidence on the intensity of plant interactions within a plant community in a given environment (Pugnaire et al., 2004; Callaway, 2007).

Most studies on plant interactions focused on the effects of a particular species on one (Callaway et al., 1996; Pugnaire et al., 1996; Sthultz et al., 2007) or several understory species (Rodríguez-Echeverría and Pérez-Fernández, 2003; Gómez-Aparicio et al., 2004; Padilla and Pugnaire, 2009; Pistón et al., 2015). In plant communities, where usually several dominant species co-exist, there is little information on how interaction outcome may vary across species (Pugnaire et al., 2004; but see Liancourt et al., 2005; Schöb et al., 2012; Pistón et al., 2016). In addition, there is a growing body of evidence suggesting that certain species are more likely to act as facilitators than others (Callaway, 2007; Paterno et al., 2016), and that these differences may be result from e.g., specific functional traits related to plant phylogeny (Garbin et al., 2014) or differential trait effect on surrounding abiotic conditions (Schöb et al., 2013a). Several mechanisms can generate species-specific and plant functional type specific facilitative relationships linked to the variety of ways facilitator species influence their environment and resources, e.g., increasing soil water or nutrient content (Callaway, 1998). For instance, several studies have found that neighboring plants growing close to leguminous species can benefit from the additional N supply to the soil, resulting in greater N concentration in their leaves (Pugnaire et al., 1996; Temperton et al., 2007). Thus, species belonging to different functional types (e.g., N-fixers and non-fixers) may differently affect performance of understory species, resulting in a range of interactions that could go from competition to facilitation within the same community (Pugnaire et al., 2004). Moreover, whether such effects are consistent at the species and community levels remains unclear (Soliveres et al., 2015).

In stressful environments, such as dry mountains, shrubs can modify the environment under their canopy by improving microclimatic conditions (Gómez-Aparicio et al., 2004; Schöb et al., 2013a) or increase availability of soil resources (Verdú and García-Fayos, 2003; Gazol and Camarero, 2012). There is also evidence that shrubs may improve conditions for the establishment and growth of other species under their canopy despite strong resource competition (Holmgren et al., 1997; Moro et al., 1997; Schöb et al., 2014a,b; Tirado et al., 2015). These mechanisms operate simultaneously (Pugnaire and Luque, 2001), exerting both positive and negative effects (e.g., improving water availability while reducing light under the canopy) that would ultimately shape plant interaction outcome. However, we have less evidence about the factors that are more important in driving plant interactions in dry mountain systems.

In the southern slopes of the Sierra Nevada Mountains in Spain, at elevations below 2500 m, precipitation is low and temperature and radiation high, leading to rather demanding conditions for plants (Callaway et al., 2002; Sánchez-Marañón et al., 2002; Schöb et al., 2013a). This system provides an opportunity to assess the contribution of different factors (e.g., water, nutrients, light or temperature) to plant interaction outcome. The co-occurrence of several shrub species with contrasting functional types (e.g., N fixers and non-fixers) makes this system suitable to address species-specific effects on understory species.

Our aim was to assess the effects of shrubs of contrasting functional type on plant interaction outcome and how limitation of multiple resources (e.g., light, water) modulates plant interactions at the single-species and community levels. We selected four dominant shrub species belonging to two different functional types, two Fabaceae, *Cytisus galianoi* and *Genista versicolor*, and two non-N-fixing species, *Bupleurum spinosum* (Apiaceae) and *Hormathophylla spinosa* (Brassicaceae), and assessed shrub effects on performance of a focal understory species, *Euphorbia nicaeensis*, using a functional trait approach (Violle et al., 2007). We additionally estimated shrub effects on the herbaceous plant community beneath shrubs, recording the number of species and of individuals per species. Hence we tested whether effects at the species level paralleled effects at community level, and whether these effects were consistent across different shrub species and functional types. We expected that shrubs from different functional type would differ in their effects on *Euphorbia* performance and the understory community, and that species-specific effects on *Euphorbia* would be scalable to the whole community.

## Materials and Methods

### Field Site and Shrub Species

Our field site was in the southern slope of the Sierra Nevada Mountains, South–East Spain. The field site is at 2040 m elevation (36°57′52″N; 03°20′12″W), slightly below the tree line. The site is dominated by shrub species like *Cytisus galianoi, Genista versicolor*, or *Hormathophylla spinosa* (Valle, 2003). The soil is an Inceptisol belonging to the Ochrept suborder (de la Rosa et al., 2001). The bedrock is micha-schist, which determines the gentle hillsides found in the Sierra Nevada Mountains (Delgado et al., 2001). The climate is dry continental Mediterranean with a hot and dry summer (means of 17°C and 5 mm rainfall in July at Pradollano, 2500 m elevation), with 30% of the precipitation in form of snow above 1800 m^1^. Mean annual rainfall is ∼800 mm and mean annual temperature is 6°C (Delgado et al., 2001). The growing season extends from June to September with peak in July.

The study was conducted on a west-oriented ∼1 ha plot with 14.8% slope where we selected four shrubs species including two N-fixing species, *Cytisus galianoi* Talavera and Gibbs (Fabaceae) and *Genista versicolor* Boiss. (Fabaceae), and two non-N-fixing species, *Bupleurum spinosum* Gouan (Apiaceae) and *Hormathophylla spinosa* (L.) P. Küpfer (Brassicaceae), all common in the Sierra Nevada Mountains. Total shrub cover (a mix of species) was approximately 80% with the remaining surface covered by open areas. The cover was an order of magnitude lower for *Bupleurum spinosum* and *Hormathophylla spinosa* shrubs as compared to *Cytisus galianoi* and *Genista versicolor*. The understory of our four shrub species and open areas were mainly colonized by small perennial herbs and grasses (**Supplementary Table S1**).

### Focal Understory Species

We selected as target a fairly common species, *Euphorbia nicaeensis* All. ssp. *nicaeensis* (Euphorbiaceae), which grows often associated with shrubs but also in open areas. *Euphorbia* is a forb common from sea level to 2400 m elevation that grows on ruderal and nitrofilous habitats generally on calcareous substrates at sunny places (Benedí et al., 1997). We selected *Euphorbia* individuals growing under each of the four dominant shrub species and in open areas (between shrubs), with 11 replicates per microhabitat.

### Abiotic Conditions

We recorded photosynthetically active radiation (PAR), volumetric soil water content (SWC), relative air humidity (RH) and air temperature under individual shrubs of each species and in open areas. PAR was measured by using integrated paper sensors (Padilla et al., 2011) placed at ground level next to *Euphorbia* individuals in all microhabitats and left for five consecutive days in the field. Integrated paper sensors consist of several small booklets of light sensitive sheets (between 9 and 12) placed in black envelopes with a circular aperture ∼2 cm in diameter that allows light exposure on the photosensitive paper face. Sheets were then exposed to dry ammonia vapors for stabilization. After calibration (Padilla et al., 2011), mean daily PAR values were estimated from the number of bleached sheets.

Soil samples of the top 10 cm were collected next to *Euphorbia* individuals on July 2011, and immediately weighed to obtain fresh mass. Then, samples were oven-dried at 105°C for 72 h until constant weight, and re-weighed to obtain dry mass. SWC was obtained by dividing the difference in mass between fresh and dry samples by soil volume. Since SWC was measured on a single date, in order to show long-term differences in soil moisture among shrubs and between shrubs and open areas, we recorded soil moisture under shrubs (*n* = 3 for each species except *Bupleurum spinosum*) and in open areas (*n* = 3) using ECH_2_O probes connected to Em50 dataloggers (Decagon Devices, Pullman WA, USA) between May and October; Air temperature and relative humidity (RH) were recorded for the same period using iButtons (Maxim Integrated Products, Sunnyvale, CA, USA) placed within shrubs (*n* = 3 for each species) and in open areas (*n* = 3).

### Plant Functional Traits

We assessed *Euphorbia* performance through traits (**Table 1**) measured following standard protocols (Cornelissen et al., 2003; Pérez-Harguindeguy et al., 2013). We measured (i) specific leaf area (SLA); (ii) leaf dry matter content (LDMC); (iii) relative water content (RWC); (iv) quantum efficiency (ΔF/F_M_′) of photosystem II (PSII); (v) photosynthetic rate (A); (vi) stomatal conductance (g_s_), (vii) transpiration rate (E), (viii) leaf carbon (C) and (ix) leaf nitrogen (N) contents. SLA and LDMC were determined using 8-10 leaves per individual. Fully developed leaves were collected between 8 and 10 am from vegetative stems, rehydrated under dark conditions for 24 h and weighed (FW_sat_). Leaves were then oven dried at 70°C (DW) and weighed again. Fresh leaves were scanned at 300 dpi and the projected leaf area (LA) measured using image analysis software (Midebmp v.4.2, Almería, Spain). SLA was then calculated as SLA = LA/DW; LDMC was calculated as LDMC = DW/FW_sat_ and the relative water content was calculated as RWC = ((FW – DW)/DW)*100. Leaves were homogeneously ground using a ball mill and C and N contents determined with an elemental analyzer (CHN model EA 1108; Carlo Erba Instruments, Milan, Italy) at the Chemical Analysis Facility at CEFE (Montpellier, France).

**Table 1:** Functional, growth and reproductive plant traits of *Euphorbia nicaeensis* individuals and abiotic variables under four shrub species (*Genista versicolor*, *Cytisus galianoi*, *Hormathophylla spinosa* and *Bupleurum spinosum*) and in open areas.

		*Cytisus*	*Genista*	*Bupleurum*	*Hormathophylla*	Open area
**Functional traits**						
A (n.s.)	Photosynthesis rate (μmol m^-2^ s^-1^)	3.77 ± 0.38	4.26 ± 0.66	4.02 ± 0.55	3.51 ± 0.66	4.01 ± 0.63
E (n.s.)	Transpiration rate (mmol m^-2^ s^-1)^	0.25 ± 0.05	0.21 ± 0.04	0.24 ± 0.03	0.21 ± 0.04	0.24 ± 0.04
gs (n.s.)	Stomatal conductance (mmol m^-2^ s^-1^)	11.36 ± 2.13	9.53 ± 1.75	11.22 ± 1.21	9.52 ± 2.00	10.83 ± 1.74
WUE (n.s.)	Instantaneous water use efficiency (μmol CO_2_ m^-2^ s^-1^ mol^-1^ H_2_O m^-2^ s^-1^)	18.94 ± 2.42	21.97 ± 1.75	18.50 ± 2.84	21.31 ± 2.7	18.75 ± 2.24
ΔF/F_m_′ (n.s.)	ΔF/F_m_′	0.26 ± 0.04	0.20 ± 0.03	0.27 ± 0.05	0.22 ± 0.04	0.33 ± 0.05
SLA (n.s.)	Specific leaf area (mm^2^ mg^-1^)	140.98 ± 15.48	115.70 ± 5.83	116.56 ± 8.24	114.80 ± 6.81	103.85 ± 7.26
LDMC (n.s.)	Leaf dry matter content (mg g^-1^)	312.63 ± 8.91	319.45 ± 10.91	332.70 ± 12.48	326.97 ± 10.49	344.33 ± 11.22
RWC (n.s.)	Leaf relative water content (%)	80.03 ± 1.24	82.06 ± 1.44	78.07 ± 1.03	79.59 ± 1.72	80.89 ± 2.34
C^∗^	Leaf C content (mg g^-1^)	43.72 ± 0.19a	43.80 ± 0.14a	39.71 ± 3.77ab	43.51 ± 0.18ab	42.92 ± 0.25b
N^∗∗∗^	Leaf N content (mg g^-1^)	1.93 ± 0.10a	1.99 ± 0.08a	1.59 ± 0.09bc	1.69 ± 0.08abc	1.43 ± 0.09c
C/N^∗∗∗^	Leaf C to N ratio	23.29 ± 1.18bc	22.37 ± 0.90c	28.86 ± 1.59ab	26.46 ± 1.32abc	30.90 ± 1.62a
**Growth and reproductive traits**						
Plant height^∗^	Height of vegetative stems (cm)	25.58 ± 0.99a	23.27 ± 1.43ab	21.94 ± 1.60ab	20.18 ± 1.56b	18.45 ± 1.61b
Vegetative stems (n.s.)	Number of vegetative stems per individual	3.42 ± 0.40	4.00 ± 0.43	2.71 ± 0.45	3.64 ± 0.54	4.70 ± 0.99
Reproductive stems^∗∗^	Number of reproductive stems per individual	3.08 ± 1.43a	1.64 ± 0.51a	2.09 ± 0.91a	2.45 ± 1.01a	12.20 ± 3.48b
Infrutescence volume^∗∗^	Volume of the infrutescence in reproductive stems (cm^3^)	2.26 ± 0.67ab	1.21 ± 0.53a	1.37 ± 0.49a	0.49 ± 0.17a	4.14 ± 1.06b
Seed mass (n.s.)	Mean seed mass (mg)	0.73 ± 0.23	0.70 ± 0.31	0.91 ± 0.27	0.80 ± 0.29	1.26 ± 0.23
Total seeds^∗^	Total number of seeds per individual	60.59 ± 6.43a	52.09 ± 37.66a	62.21 ± 37.94a	44.45 ± 24.62a	182.1 ± 9.87b
**Abiotic variables**						
PAR^∗∗∗^	Photosynthetically active radiation (μmol m^-2^ s^-1^)	361 ± 39.53a	344 ± 37.88a	240 ± 54.35a	267 ± 46.12a	1328 ± 62.59b
SWC^∗∗∗^	Volumetric soil water content (cm^3^ cm^-3^)	2.72 ± 0.25ab	4.03 ± 0.78a	1.62 ± 0.14bc	2.35 ± 0.23bc	0.87 ± 0.11c
RH^∗∗∗^	Air relative humidity (%)	60.25 ± 2.24a	63.42 ± 2.24a	NA	66.10 ± 2.22a	50.65 ± 2.02b
T^∗∗∗^	Air temperature (°C)	16.05 ± 0.49a	17.04 ± 0.52a	NA	16.26 ± 0.45a	19.03 ± 0.57b


*Values are mean ± SE. Results of overall test are shown in parentheses: n.s., non significant, ^∗^*P* < 0.05, ^∗∗^*P* < 0.01, ^∗∗∗^*P* < 0.001. Different letters indicate significant differences between shrubs or with open areas (Tukey HSD tests; *P* < 0.05).*

Gas exchange parameters (A, g_s_ and E) were measured *in situ* using a portable system (LI 6400; Li-COR, Lincoln, Nebraska, USA) under constant conditions (PAR = 1500 μmol m^-2^ s^-1^; RH = 40%; T_leaf_ = 25°C) at ambient CO_2_. Each time 3–5 leaves were kept in the leaf chamber for stabilization and gas exchange recorded at 3-min intervals. Instantaneous water use efficiency (WUE) was then calculated as WUE = A/E. Chlorophyll fluorescence (ΔF/F_M_′) was measured using a photosynthesis yield analyzer (MINI-PAM; Heinz Walz GmbH, Effeltrich, Germany) under ambient radiation and steady-state conditions. All gas exchange and chlorophyll fluorescence traits were measured along 4 days between 8 am and 12 am under cloudless conditions.

Plant size was calculated as S = H^∗^V where H is plant height and V is the number of vegetative stems. We tested the relationship between S and biomass on a subset of 31 individuals collected within and outside shrubs in the same field site; both variables were positively correlated (*r* = 0.95, *p* < 0.001, *n* = 31), similar to data obtained in other species elsewhere (Prieto et al., 2011; de Bello et al., 2012). We then used plant size as a surrogate for plant biomass to avoid destructive measurements. To assess interaction intensity, the relative interaction index (RII; Armas et al., 2004) was calculated as RII = (S_in_ – S_op_)/(S_in_ + S_op_); where S_in_ and S_op_ are plant size under shrubs and in open areas respectively.

The number of reproductive stems and the ratio of vegetative-to-reproductive stems were determined for each *Euphorbia* individual. Infrutescence volume (i.e., the volume of the reproductive plant parts containing the seeds) was calculated as a spherical ellipsoid based on the two diameters measured on the horizontal plane orthogonal to the reproductive stem. In July 2011, reproductive stems of *Euphorbia* individuals were bagged with nylon mesh to prevent seed loss; in September 2011, once all seeds were estimated to be mature, seeds contained in the mesh bags were collected, counted and dried for 72 h at 60°C. The number of reproductive stems, number of seeds and mean seed mass were used as surrogates of plant fitness.

### Community-Level Data

We recorded the number of understory species and individuals by sampling 30 quadrats under each shrub species and 120 in open areas (**Supplementary Table S1**). For the largest shrubs, *Bupleurum spinosum* (canopy area of 4992 ± 515 cm^2^; mean ± SE), *Cytisus galianoi* (8898 ± 947 cm^2^) and *Genista versicolor* (7290 ± 1298 cm^2^) we sampled one 25 cm × 25 cm quadrat randomly placed within the canopy, and for the smallest shrub, *Hormathophylla spinosa* (2452 ± 263 cm^2^), we sampled the whole area beneath the canopy. A quadrat of the same size (25 cm × 25 cm, or size of the whole canopy in case of *H. spinosa*) was randomly sampled in a nearby open area (at least 1 m away from the shrub). Measurements were done at the peak growing season (mid July 2011).

### Statistical Analyses

We used redundancy analysis (RDA; Canoco 4.5 package; ter Braak and Šmilauer, 2002) to assess correlation between *Euphorbia* traits in different microhabitats. RDA provides a good estimate of main trends in the data and can be considered an extension of multivariate regression for a multivariate response variable (Lepš and Šmilauer, 2003). The parametric test is replaced by Monte Carlo permutations to overcome problems with distributional characteristics. We used the forward selection procedure to identify the main environmental characteristics explaining a significant part of the variation in *Euphorbia* traits. First, we assessed the global significance of the model. Second, we used partial analyses (i.e., we included the tested factor as the only explanatory variable and the other factors as covariates) to obtain the variability explained by each individual factor and their corresponding significance (*P*-value). Tests were based on 499 random permutations. The ordination diagram as an output of the RDA shows linear correlations between quantitative and qualitative environmental variables (arrows and points, respectively) and measured *Euphorbia* traits (arrows).

We tested differences in environmental conditions and in functional traits of *Euphorbia* growing under the different shrub species and in open areas using one-way ANOVA and subsequent *post hoc* Tukey HSD and/or Dunnett tests. We performed a second comparison to test for differences between functional types by grouping plants under N-fixing and non-N-fixing species. ANOVA assumptions of equal variance were checked using a Levene’s test and normality of the residuals using a Shapiro–Wilk test (Shapiro and Wilk, 1965); when data were not normal, non-parametric Kruskall–Wallis analyses were used followed by single pair comparisons. To avoid type I error, pair-wise comparisons between treatments for Kruskall–Wallis tests were followed by Bonferroni corrections adjusted according to Legendre and Legendre (1998). When analyzing RII, we performed Student *t*-tests against a constant value (zero). Positive RII values significantly different from zero indicate net positive effects (i.e., facilitation) and negative, significantly different from zero indicate net negative effects (i.e., competition). When not significant, net effects were neutral (i.e., partial positive and negative effects have the same magnitude). Long-term soil moisture below shrubs and in open areas was compared using a general linear mixed model (GLMM) with *species* (including open areas) and *time* as fixed factors and individual shrubs (or individual open areas) as the random factor. GLM, ANOVA, Kruskall–Wallis and GLMM analysis were performed using R version 2.15.3 (R Development Core Team, 2013).

We tested for differences between microhabitats (species and functional types) regarding number of individuals per square meter in the understory using Wilcoxon matched pairs tests. Each shrub species was compared with their paired open area due to different plot sizes for big and small shrubs.

## Results

**FIGURE 1:**
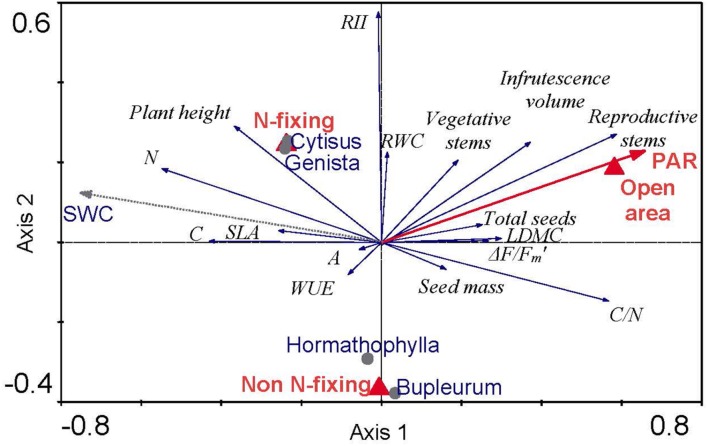
**Redundancy analysis, RDA, triplot ordination with recorded *Euphorbia* traits and model selected environmental conditions (PAR and Microhabitat type: open area and N-fixing).** Response variables (in *italics*) are indicated by arrows and labeled as follows: *N*, leaf nitrogen content; *C*, leaf carbon content; *SLA*, specific leaf area; *WUE*, water use efficiency; *A*, photosynthetic rate; *RII*, relative interaction index; *RWC*, relative water content; *LDMC*, leaf dry matter content; *C/N*, leaf C to N ratio. The environmental variables are indicated by a bold red arrow for continuous variables (PAR) and triangles in the case of categorical variables (Open area, N-fixing and Non N-fixing species). Centroids of species identity (circles) and soil water (dashed line) are added as supplementary variables without any effect to the analyses. The first canonical axis explains 11.1% of variability (*F* = 6.39, *P* = 0.002) and all three canonical axes explain 18.4% of variability (*F* = 3.83, *P* = 0.002). The explanatory variables Open area, N-fixing and PAR were selected by forward selection procedure (all at *P* < 0.05) but the variable Non N-fixing is also shown to help interpretation.

Redundancy analysis showed significant conditional effects of three environmental characteristics on *Euphorbia* growth, one continuous variable (PAR), and two categorical variables related to species identity, open areas and N-fixing ability (**Figure 1**). These environmental traits altogether explained 18.4% of variance. Partial effects of all selected explanatory variables were highly significant (Open areas, *P* = 0.002; Functional type, *P* = 0.004; PAR, *P* = 0.024). These variables explained 5.5, 5.0, and 3.2% of variance in our dataset, respectively. The first ordination axis was positively correlated with PAR and tell apart *Euphorbia* individuals according to their position under shrubs (negative values) versus open areas (positive values). All reproductive traits (number of reproductive stems, infrutescence volume, total seeds and seed mass) were positively correlated to this first axis; similarly, ΔF/Fm’, LDMC and C/N were also positively correlated to PAR and open areas (Axis 1). On the other hand, nitrogen content (N), SLA and plant height were negatively correlated to the first axis of variation and were thus negatively correlated to PAR. Individually, LDMC was negatively correlated to leaf N, whereas SLA was positively correlated to leaf N. The second ordination axis shows differences between N-fixing and non-N-fixing shrub species. RWC and RII were positively correlated to this axis of variation, both being greater in *Euphorbia* individuals growing under N-fixing shrubs. Similarly, plant height, the number of vegetative and reproductive stems, and leaf N tended to be positively correlated to N-fixing shrubs. Finally, there was no correlation between A or WUE to any of these two axes.

### Abiotic Conditions

**FIGURE 2:**
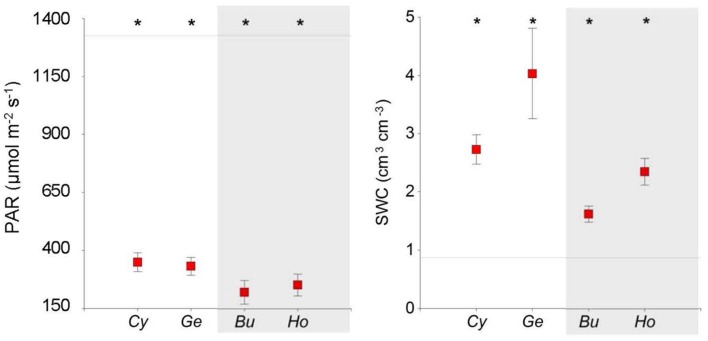
**Soil water content (SWC) and mean daily photosynthetically active radiation (PAR) measured under four shrub species and in open areas in the southern slope of the Sierra Nevada Mountains.** Mean values ± SE are shown. Asterisks indicate differences with open areas (Dunnett test, *P* < 0.05). Horizontal lines represent mean values for open areas. *Cy*, *Cytisus galianoi*, *Ge*, *Genista versicolor*, *Bu*, *Bupleurum spinosum*, *Ho*, *Hormathophylla spinosa*. Shaded area includes non-N-fixing species.

Soil water content was higher and PAR lower under shrubs than in open areas (**Table 1**, **Figure 2**), and both SWC and PAR were greater under N-fixing than under non-N-fixing shrubs (**Table 2**). Microhabitats differed in mean RH (*F* = 17.51, *P* < 0.001) and air temperature (*F* = 5.60, *P* < 0.023). Mean air temperature was higher and RH was lower in open areas than under shrubs (**Table 1**; **Supplementary Figure S1**). Long-term soil moisture was lower in open areas than under *Hormathophylla* or *Genista*, with *Cytisus* showing intermediate values (*F* = 2.73, *P* < 0.001).

**Table 2:** ANOVA (regular font) and *Kruskall–Wallis (Italics)* results for plant growth, ecophysiological traits, and continuous environmental variables of *Euphorbia nicaeensis* individuals growing in contrasting microhabitats under the N-fixing shrub species (*Genista versicolor, Cytisus galianoi*) versus non-N-fixing species (*Hormathophylla spinosa, Bupleurum spinosum*) in the southern slope of the Sierra Nevada Mountains (Granada, Spain).

	F or χ^2^	*P*-value
A	0.18	0.675
E	0.02	0.878
gs	0.00	0.966
WUE	0.04	0.844
ΔF/F_m_′	0.24	0.625
*SLA*	*0.58*	*0.447*
LDMC	1.75	0.192
RWC	2.61	0.114
C	1.78	0.189
N	**13.30**	**0.001**
C/N	**12.84**	**0.001**
Plant height	**3.95**	**0.050**
*Vegetative stems*	*1.44*	*0.230*
*Reproductive stems*	*0.00*	*0.972*
*Infrutescence volume*	*1.32*	*0.250*
Seed mass	1.07	0.350
*Total seeds*	*0.11*	*0.739*
PAR	**5.72**	**0.021**
SWC	***6.70***	***0.001***


*Variables are named according to acronyms in **Table 1**. Bold indicates significant differences between N-fixing and non-N-fixing shrubs (*P* ≤ 0.05).*

### Plant Functional Traits

**FIGURE 3:**
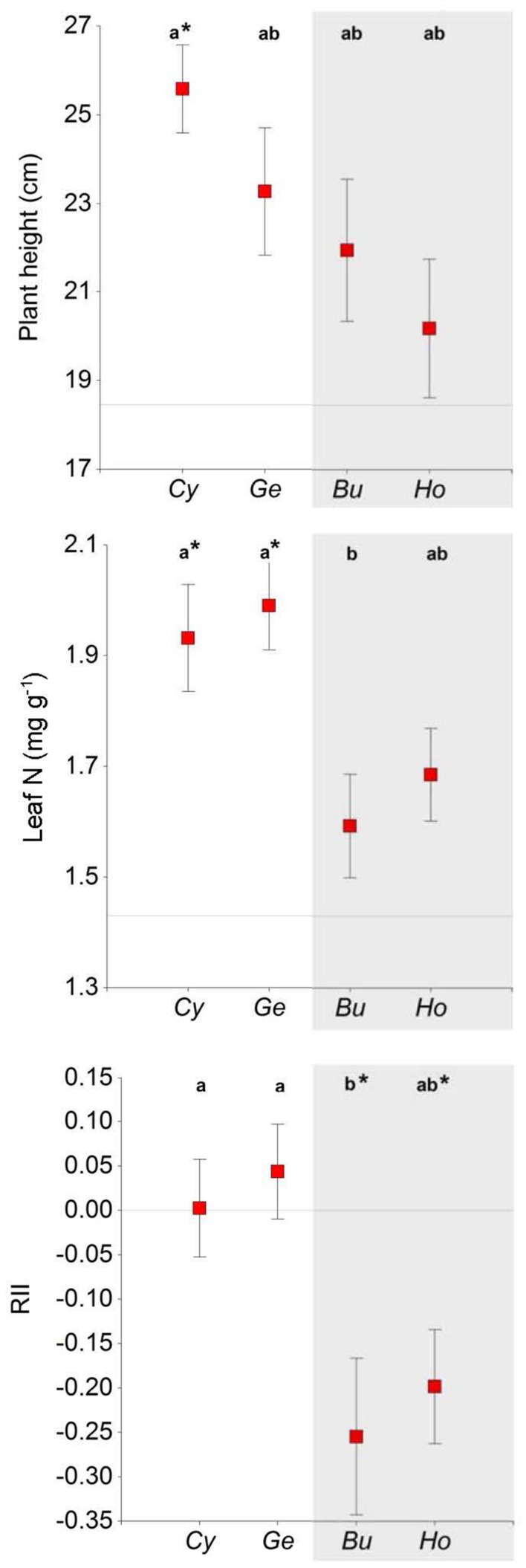
**Plant height, leaf N content, and relative interaction index (RII) in *Euphorbia nicaeensis* plants growing under four shrub species and in open areas in the southern slope of the Sierra Nevada Mountains.** Mean values ± SE are shown. Different letters indicate significant differences between shrubs (Tukey HSD tests; *P < 0.05*), and asterisks indicate differences with open areas (Dunnett test, ^∗^*P < 0.05*). Horizontal lines represent mean values for open areas; note that RII = 0 in open areas by definition, in this case asterisks indicate RII is significantly different from zero (*t*-test, *P* < 0.01). *Cy*, *Cytisus galianoi*, *Ge*, *Genista versicolor*, *Bu*, *Bupleurum spinosum*, *Ho*, *Hormathophylla spinosa*. Shaded area includes non-N-fixing species.

*Euphorbia* leaf traits (i.e., LDMC and SLA) did not differ among shrubs, between shrubs and open areas (**Table 1**), or between functional types (**Table 2**). Organic C and total N contents were higher in *Euphorbia* individuals growing under shrubs than in gaps (**Table 1**; **Figure 3**). Leaf N was also higher in *Euphorbia* individuals growing under N-fixing than under non-N-fixing shrubs (**Table 2**; **Figure 3**). Neither shrub species nor functional type had an effect on the physiological status of *Euphorbia*; A, E, gs, WUE, ΔF/F_m_′ and RWC did not differ among microhabitats (species or open areas, **Table 1**), nor between N-fixing and non-N-fixing shrubs (**Table 2**).

*Euphorbia* individuals were taller under *Cytisus* than in open areas (**Figure 3**; **Table 1**), and also under N-fixing than under non-N-fixing species (**Figure 1**; **Table 2**). The relative interaction index (RII) was greater under N-fixing than under non-N-fixing shrubs (*F* = 14.58, *P* < 0.001), and did not differ from zero in N-fixing species (*Genista* and *Cytisus*) indicating neutral effects, while it was significantly negative in non-N-fixing species (*Hormathophylla* and *Bupleurum*) indicating net competitive effects (**Figure 3**).

**FIGURE 4:**
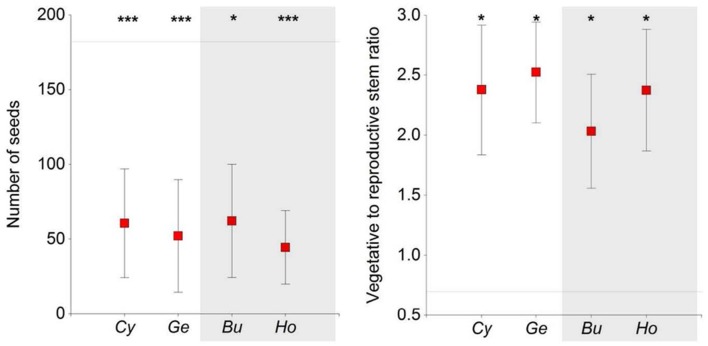
**Number of seeds produced and number of vegetative stems to number of reproductive stems (mean ± SE) of *Euphorbia* individuals under four shrub forming species and in open areas at a study site located in the southern slope of the Sierra Nevada Mountains.** The horizontal line represents the mean value for open areas. Asterisks indicate differences between shrubs and open areas (Dunnett test, ^∗^*P* < 0.05, ^∗∗∗^*P* < 0.001). *Cy*, *Cytisus galianoi*; *Ge*, *Genista versicolor*; *Bu*, *Bupleurum spinosum*; *Ho*, *Hormathophyllaspinosa*. Shaded area includes non-N-fixing species.

Contrasting allocation patterns were observed in *Euphorbia* individuals growing in open areas versus growing under shrubs, the former having lower vegetative-to-reproductive stem ratios (*F* = 2.54, *P* = 0.05). There were no differences in these ratios between shrub species or functional types (**Figure 4**). Furthermore, *Euphorbia* individuals growing in open areas produced more reproductive stems and had a higher number of seeds (**Table 1**; **Figure 3**) than individuals growing under shrubs regardless of shrub species or functional type. Mean seed mass, however, was similar in individuals growing under shrubs and in open areas (**Table 1**).

**FIGURE 5:**
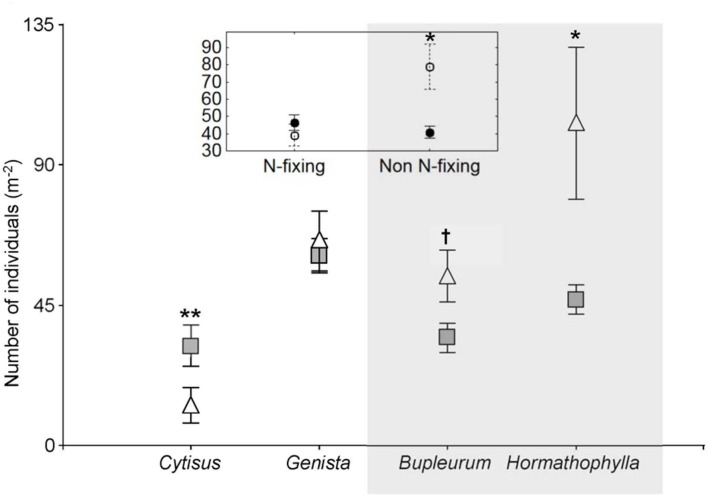
**Number of individuals per m^2^ (mean ± SE) under four shrub forming species (full symbols) and in open areas (open symbols), and (inset graph) between two functional types at a study site located in the southern slope of the Sierra Nevada Mountains (Granada, Spain, 2040 m elevation).** Shrub forming species are *Cytisus galianoi*, *Genista versicolor*, *Bupleurum spinosum*, and *Hormathophylla spinosa*. Shaded area includes non-N-fixing species. Significant differences between shrubs and respective open areas are marked: ^∗∗^*P* < 0.01; ^∗^*P* < 0.05; ^†^*P* < 0.1.

At the community level, the four shrub species had contrasting effects on the number of individuals growing in the understory. While *Cytisus galianoi* acted as a facilitator (*Z* = 2.13, *P* = 0.033), the effect of *Genista versicolor* was neutral (Z = 0.19, *P* = 0.85), and *Bupleurum spinosum* and *Hormathophylla spinosa* acted as competitors –although with different intensities (*Z* = 1.91, *P* = 0.05, and *Z* = 1.96, *P* = 0.049, respectively) (**Figure 5**). When analyzed by functional type, N fixing species exerted neutral effects (Z = 1.25, *P* = 0.21) whereas non-N-fixing species exerted net competitive effects (Z = 2.65, *P* = 0.008), (**Figure 5**).

## Discussion

Our data show specific effects of each shrub species on its understory community, although functional type (N-fixing vs. non-N-fixing shrubs) was a reasonably good predictor of interaction outcome. The effects of each species were consistent at the species and community levels, and were a consequence of their functional type; although we did not measure individual shrub traits, the two non-legume shrubs exerted a net competitive effect on *Euphorbia* (species level) and on the number of individuals growing beneath them (community level) whereas the two legume shrubs presented neutral effects regardless of level. Finally, *Euphorbia* displayed a high phenotypic plasticity in response to the shrub presence.

We did not find net facilitation effects but rather evidence of strong competition (under non-N-fixing shrubs) or a partial release of competition under N-fixing shrubs (Holmgren et al., 1997; Maestre et al., 2003). At the species level, this was most evident regarding biomass, as RII was neutral under N-fixing shrubs (*Cytisus* and *Genista*) and negative under non-fixing shrubs (*Bupleurum* and *Hormathophylla*). At community level we recorded net competition (lower number of subordinate individuals) under *Bupleurum* and *Hormathophylla*, a neutral effect under *Genista*, and a facilitative effect under *Cytisus*. We should note that facilitative and competitive effects are species-specific and depend strongly on the species under study (Pugnaire et al., 2004; Liancourt et al., 2005; Callaway, 2007; Padilla et al., 2009); however, the fact that these effects were similar at the species (e.g., for *Euphorbia* individuals) and community levels (number of individuals per m^2^) suggest that these results are scalable, as suggested already by Schöb et al. (2013b). This parallelism in the effects at the species and community levels suggests that using a single focal species provides reliable evidence on the processes shaping the plant community.

The contrasting allocation patterns recorded in *Euphorbia*, along with strong correlations between PAR, SWC and reproductive traits, suggest different strategies of individuals growing under shrubs and in open areas. Plants under shrubs invested relatively more in growth at the expenses of reproduction, as reported for *E. terracina* by Riordan et al. (2008). These differences in biomass allocation point also to the species considerable phenotypic plasticity and its ability to cope with stress meant by high light and low water availability. Since *Euphorbia* seeds can germinate under both light and dark conditions (Narbona et al., 2006), seeds would germinate easily under shrubs and individuals would grow better thanks to the high water and nutrient availability. Individuals in open areas, however, must cope with drier soils, higher temperatures and lower soil nutrient content, investing relatively more into reproductive organs likely to compensate for higher juvenile or adult mortality (Al Samman et al., 2001). Although reproductive allocation has a genetic basis, it can greatly vary within species depending on environmental conditions (Karlsson and Méndez, 2005; Castellanos et al., 2014). Therefore, differences in allocation patterns are *Euphorbia nicaeensis’* phenotypic responses to environmental variability under shrubs and in open areas. As the number of vegetative stems, a good index of plant age in *Euphorbia nicaeensis* (Narbona, 2002), was similar among microhabitats, we assume a similar age for all selected individuals ruling out age driven differences on the reproductive outputs observed.

The prevalence of competitive effects has been also reported for other *Euphorbia* species under dry conditions (e.g., Rinella and Sheley, 2005). Improvement of soil water availability and soil nutrients under perennial species is a major source of facilitation in semi-arid systems (Pugnaire et al., 2011). Yet, given the interaction outcome in *Euphorbia*, regarding plant size (biomass) and plant fitness (reproductive traits), and the preference of the species for sunny places (Benedí et al., 1997), it appears that light is the limiting factor for this species (cf. Grime, 1973). Our data support this idea since reproductive output (and reproductive-to-vegetative biomass ratio) was greater in open areas than under shrubs, despite lower soil water and nutrient content. *Euphorbia* individuals grew taller under shrubs than in open areas, which are a common response to light limitation (Macek and Lepš, 2003, 2008). Nevertheless, as height of *Euphorbia* individuals also differed between shrub microhabitats and did not correlate with PAR under shrubs (*P* = 0.90), it suggests there was no etiolation but rather an increase in plant size.

Although light is an important factor driving plant interactions (Holmgren et al., 2012), facilitation through increased nutrient availability is also common in semi-arid environments, especially by N under legume species (Pugnaire et al., 1996; Reynolds et al., 1999; Gómez-Aparicio et al., 2004; Armas et al., 2008). In our case, despite strong competition for light, *Euphorbia* benefited from improved N content and higher soil moisture under leguminous shrubs, as changes in leaf N positively correlated to plant height and size (i.e., taller plants and greater RII under N-fixing shrubs). Hence, the lower competition intensity observed under N-fixing shrubs was most probably a consequence of increased resource availability. This benefit may be, however, difficult to separate from improved soil water conditions, as plant N and water uptake are usually tightly linked (Hu and Schmidhalter, 2005; Macek et al., 2009).

Recent climate models predict an increase in aridity and temperature in Southern Europe (IPCC, 2007), and particularly under Mediterranean climate, where drier conditions would be found at higher elevations as compared to current conditions (Ackerly et al., 2010). Hence, plant association patterns could be altered by increased competition between shrubs and beneficiary species (Schöb et al., 2014a). An understanding of the mechanisms that drive these interactions could help forecast future changes in structure, function and assembly of plant communities under changing climate (Callaway, 2007). We hypothesize that a competitive displacement of some shrub species and a change in shrub community structure with increasing environmental harshness may alter plant interaction patterns in dry mountain systems, leading to a community structure where the ratio between N-fixing and non-N-fixing shrubs will play an important role in the Sierra Nevada Mountains.

## Author Contributions

PM, IP, and JM conceived the study. PM, IP, JM, and NP performed field data collections. PM and IP analyzed the data and PM, IP, and FP wrote the manuscript.

## Conflict of Interest Statement

The authors declare that the research was conducted in the absence of any commercial or financial relationships that could be construed as a potential conflict of interest.

The reviewer JN declared a past co-authorship with one of the authors IP to the handling Editor, who ensured that the process met the standards of a fair and objective review.

## Footnotes

1 http://www.magrama.gob.es/es/red-parques-nacionales

## Supplementary Material

**FIGURE S1 | Air relative humidity in open areas (*n* = 3) and below shrubs (*n* = 3 for each shrub species) recorded during the season 2011.** Mean weekly data ± 95% cv are shown. Different letters indicate significant differences between microsites (open areas and shrub species; Tukey HSD tests; *P* < 0.05).

**TABLE S1 | List of understory species found below shrubs and in open areas and the number of individuals of each species in each microsite (under shrubs and open areas).** The four shrub species are depicted in bold, and total numbers of subordinate species growing beneath them are in brackets. Table modified from Pistón et al. (2016).
