# Supplementary figures and images for: Functional Plant Types Drive Plant Interactions in a Mediterranean Mountain Range
Source: Front Plant Sci. 2016 May 23;7:662. doi: 10.3389/fpls.2016.00662 (PMC4876123; doi:10.3389/fpls.2016.00662)

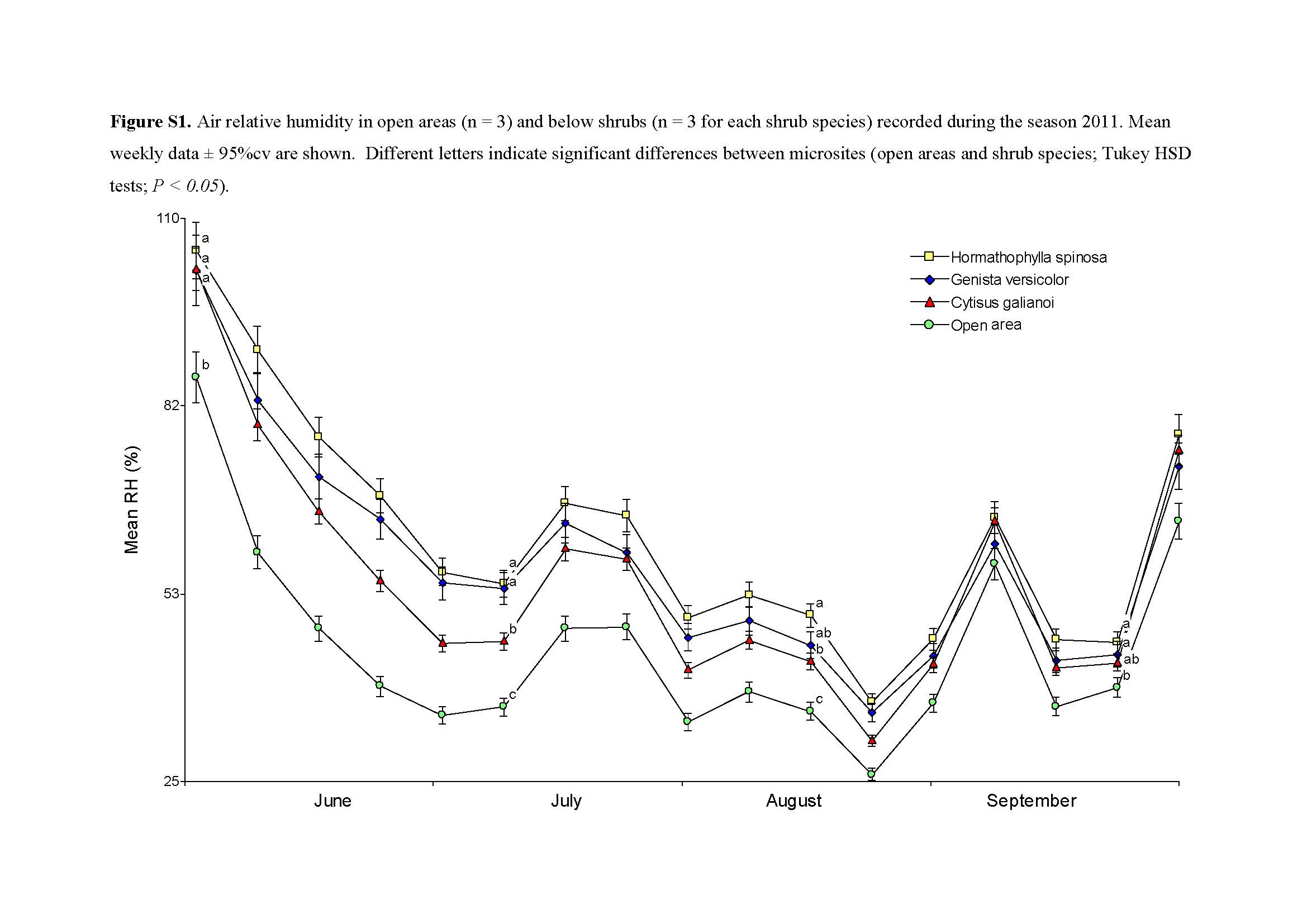

Supplement: Supplementary file 1 [file Image_1.JPEG]
