# Supplementary material for: Functional Plant Types Drive Plant Interactions in a Mediterranean Mountain Range
Source: Front Plant Sci. 2016 May 23;7:662. doi: 10.3389/fpls.2016.00662 (PMC4876123; doi:10.3389/fpls.2016.00662)
Supplement: Supplementary file 2 [file Table_1.DOCX]

**Table S1.**

| \| **Species** \| **Under shrubs** \| **Open areas** \| \| --- \| --- \| --- \| \| *Alyssum nevadense* \| 0 \| 2 \| \| *Agrostis nevadensis* \| 2 \| 0 \| \| *Andryala ragusina* \| 7 \| 0 \| \| *Arenaria grandiflora* \| 2 \| 0 \| \| *Artemisia chamaemelifolia* \| 4 \| 3 \| \| *Asperula aristata* \| 4 \| 0 \| \| *Avenella flexuosa* \| 2 \| 11 \| \| *Avenula bromoides* \| 18 \| 1 \| \| *Bromus tectorum* \| 11 \| 96 \| \| ***Bupleurum spinosum* (8)** \| 22 \| 6 \| \| *Campanula lusitanica* \| 5 \| 4 \| \| *Carduus carlinoides* \| 6 \| 3 \| \| *Centaurea sp* \| 9 \| 0 \| \| *Cerastium gibraltaricum* \| 10 \| 9 \| \| *Cuscuta triumvirati* \| 5 \| 0 \| \| ***Cytisus galianoi* (13)** \| 20 \| 13 \| \| *Dianthus brachyanthus* \| 10 \| 4 \| \| *Eryngium bourgatii* \| 6 \| 19 \| \| *Euphorbia nevadensis* \| 5 \| 0 \| \| *Euphorbia nicaeensis* \| 18 \| 0 \| \| *Festuca indigesta* \| 194 \| 201 \| \| *Festuca pseudeskia* \| 5 \| 1 \| \| *Festuca sp* \| 1 \| 0 \| \| ***Genista versicolor* (13)** \| 14 \| 11 \| \| *Herniaria boissieri* \| 0 \| 5 \| \| ***Hormathophylla spinosa* (13)** \| 16 \| 2 \| \| *Jasione crispa* \| 6 \| 1 \| \| *Jurinea humilis* \| 8 \| 17 \| \| *Leontodon boryi* \| 1 \| 2 \| \| *Ononis sp* \| 4 \| 2 \| \| *Ononis spinosa* \| 15 \| 0 \| \| *Pilosella sp* \| 12 \| 0 \| \| *Poa ligulata* \| 0 \| 4 \| \| *Sanguisorba verrucosa* \| 18 \| 1 \| \| *Sedum amplexicaule* \| 0 \| 3 \| \| *Teucrium sp* \| 3 \| 2 \| \| *Thymus serpylloides* \| 59 \| 12 \| |  |  |  |
| --- | --- | --- | --- | --- | --- | --- | --- | --- | --- | --- | --- | --- | --- | --- | --- | --- | --- | --- | --- | --- | --- | --- | --- | --- | --- | --- | --- | --- | --- | --- | --- | --- | --- | --- | --- | --- | --- | --- | --- | --- | --- | --- | --- | --- | --- | --- | --- | --- | --- | --- | --- | --- | --- | --- | --- | --- | --- | --- | --- | --- | --- | --- | --- | --- | --- | --- | --- | --- | --- | --- | --- | --- | --- | --- | --- | --- | --- | --- | --- | --- | --- | --- | --- | --- | --- | --- | --- | --- | --- | --- | --- | --- | --- | --- | --- | --- | --- | --- | --- | --- | --- | --- | --- | --- | --- | --- | --- | --- | --- | --- | --- | --- | --- | --- | --- | --- | --- |
|  |  |  |  |
|  |  |  |  |
